# Human blood myeloid and plasmacytoid dendritic cells cross activate each other and synergize in inducing NK cell cytotoxicity

**DOI:** 10.1080/2162402X.2016.1227902

**Published:** 2016-09-02

**Authors:** Jasper J. P. van Beek, Mark A. J. Gorris, Annette E. Sköld, Ibrahim Hatipoglu, Heleen H. Van Acker, Evelien L. Smits, I. Jolanda M. de Vries, Ghaith Bakdash

**Affiliations:** a Department of Tumor Immunology, Radboud University Medical Center, Radboud Institute for Molecular Life Sciences, Nijmegen, the Netherlands; b Department of Oncology-Pathology, Karolinska University Hospital Solna, Karolinska Institutet, Stockholm, Sweden; c Laboratory of Experimental Hematology, Tumor Immunology Group (TIGR), Vaccine & Infectious Disease Institute (VAXINFECTIO), University of Antwerp, Faculty of Medicine and Health Sciences, Antwerp, Belgium; d Center for Cell Therapy and Regenerative Medicine, Antwerp University Hospital, Edegem, Belgium; e Center for Oncological Research (CORE), Faculty of Medicine and Health Sciences, University of Antwerp, Antwerp, Belgium; f Department of Medical Oncology, Radboud University Medical Center, Nijmegen, the Netherlands

**Keywords:** Adaptive immunity, cancer immunotherapy, crosstalk, myeloid DCs, NK cells, plasmacytoid DCs

## Abstract

Human blood dendritic cells (DCs) hold great potential for use in anticancer immunotherapies. CD1c^+^ myeloid DCs and plasmacytoid DCs (pDCs) have been successfully utilized in clinical vaccination trials against melanoma. We hypothesize that combining both DC subsets in a single vaccine can further improve vaccine efficacy. Here, we have determined the potential synergy between the two subsets *in vitro* on the level of maturation, cytokine expression, and effector cell induction. Toll-like receptor (TLR) stimulation of CD1c^+^ DCs induced cross-activation of immature pDCs and vice versa. When both subsets were stimulated together using TLR agonists, CD86 expression on pDCs was increased and higher levels of interferon (IFN)-α were produced by DC co-cultures. Although the two subsets did not display any synergistic effect on naive CD4^+^ and CD8^+^ T cell polarization, CD1c^+^ DCs and pDCs were able to complement each other's induction of other immune effector cells. The mere presence of pDCs in DC co-cultures promoted plasma cell differentiation from activated autologous B cells. Similarly, CD1c^+^ DCs, alone or in co-cultures, induced high levels of IFN-γ from allogeneic peripheral blood lymphocytes or activated autologous natural killer (NK) cells. Both CD1c^+^ DCs and pDCs could enhance NK cell cytotoxicity, and interestingly DC co-cultures further enhanced NK cell-mediated killing of an NK-resistant tumor cell line. These results indicate that co-application of human blood DC subsets could render DC-based anticancer vaccines more efficacious.

## Abbreviations

CCR7: C-C chemokine receptor type 7
CTL: cytotoxic T lymphocyte
DC: dendritic cell
GM-CSF: granulocyte-macrophage colony-stimulating factor
HLA: human leukocyte antigen
IFN: interferon
IL: interleukin
LPS: lipopolysaccharide
MFI: mean fluorescence intensity
MHC: major histocompatibility complex
MLR: mixed lymphocyte reaction
mDC: myeloid DC
NK: natural killer
ODN: oligodeoxynucleotide
PBL: peripheral blood lymphocyte
PBMC: peripheral blood mononuclear cell
pDC: plasmacytoid DC
PD-L1: programmed death-ligand 1
PMA: phorbol 12-myristate 13-acetate
poly(I:C): polyinosinic-polycytidylic acid
PI: propidium iodide
SEB: Staphylococcal enterotoxin B
TAA: tumor-associated antigen
Th: T helper
TLR: toll-like receptor
TNF: tumor necrosis factor

## Introduction

Dendritic cells (DCs) possess the ability to drive both adaptive and innate immune responses and therefore play a central role in antitumor immunity. Hence, they can be exploited for the treatment of immunogenic cancers, such as melanoma.^1,2^ DC-based vaccines consist of patient-derived DCs, which are stimulated and loaded with tumor-associated antigens (TAAs) *ex vivo*, before being re-infused into the patient. This form of immunotherapy aims at activating the patient's own immune system to recognize and eradicate tumor cells. DCs derived from monocytes or CD34^+^ progenitor cells are usually the main source of DCs used for vaccines. Nevertheless, the use of naturally occurring DCs isolated from blood has been recently hypothesized to improve therapeutic responses, due to less extensive *ex vivo* culture periods.^3^

Several DC subsets can be identified in human peripheral blood.^4^ They are divided into plasmacytoid DCs (pDCs) and myeloid DCs (mDCs), with the latter being subdivided into CD1c^+^ (or BDCA1^+^) DCs and the rare CD141^+^ (or BDCA3^+^) DCs. mDCs and pDCs are functionally distinct, which is reflected by their non-overlapping repertoire of expressed toll-like receptors (TLRs).^5-7^ Cytokine secretion following stimulation also differs between DC subsets. CD1c^+^ DCs can secrete high levels of bioactive interleukin (IL)-12,^8^ an important cytokine for the induction of T helper 1 (Th1) and cytotoxic T lymphocyte (CTL) responses.^9,10^ In contrast, pDCs can produce massive amounts of type I interferons (IFNs) upon stimulation.^11^ IFN-α can also take part in the skewing of Th1 responses,^12^ in addition to increasing the cytotoxic activity of natural killer (NK) cells.^13^ These characteristics make both DC subsets suitable for use in cancer immunotherapy. Indeed, vaccination with CD1c^+^ DCs for prostate cancer was shown to be safe and feasible.^14^ Our group has previously conducted phase I clinical trials exploiting either pDCs or CD1c^+^ DCs for vaccination of melanoma patients, demonstrating the safety and efficacy of this approach.^15,16^ These promising results raise the question whether combining both DC subsets can further improve immunological responses and clinical outcome.

In the current study, we have characterized the crosstalk between human blood CD1c^+^ DCs and pDCs by analyzing maturation status and cytokine production after co-culture of both subsets in the presence or absence of TLR stimulation. In addition, functional implications of crosstalk-mediated effects on other adaptive and innate immune cells, namely naive CD4^+^ and CD8^+^ T cells, B cells and NK cells, were addressed. We have shown that CD1c^+^ DCs and pDCs are able to cross activate each other. Although co-application of DC subsets did not augment T cell polarization, it did allow for complementation of subset-specific effector functions, including induction of plasma cell differentiation from B cells and secretion of high levels of IFN-γ by peripheral blood lymphocytes (PBLs) and NK cells. Furthermore, DC co-cultures could synergistically enhance NK cell-mediated killing of an NK-resistant tumor cell line. These results indicate that combining the distinct qualities of different human blood DC subsets may potentiate current DC-based anticancer vaccines for optimal therapeutic outcome.

## Results

### TLR triggering of human CD1c^+^ DCs or pDCs cross activates bystander DCs

**Figure 1.**
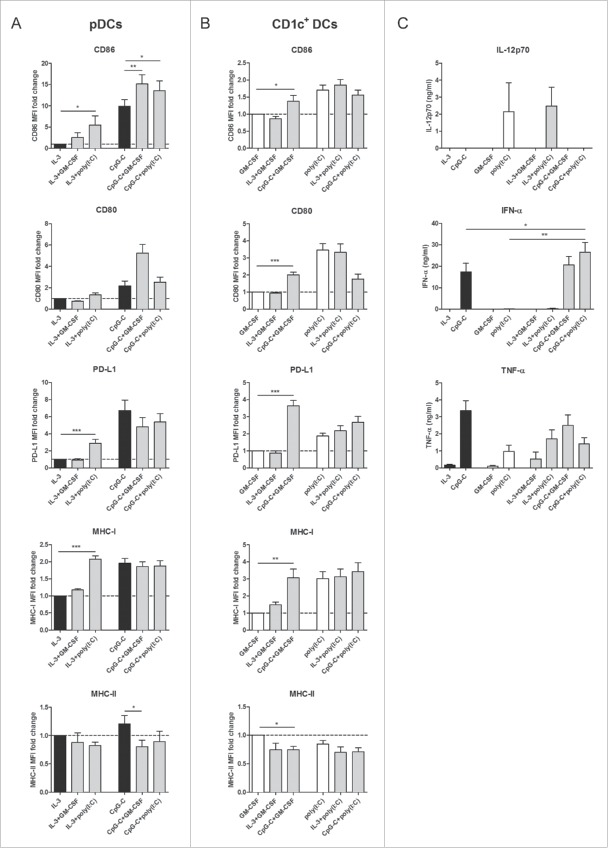
Human CD1c^+^ DCs and pDCs cross activate each other. CD1c^+^ DCs and pDCs were cultured overnight, either separately or together, in the presence of indicated stimuli. Maturation was checked on the specific DC subsets within the co-culture. (A) Relative expression of CD86, CD80, PD-L1, and MHC class I and II on pDCs in geometric MFI normalized to pDCs cultured alone with IL-3. (B) Relative expression of CD86, CD80, PD-L1 and MHC class I and II on CD1c^+^ DCs in geometric MFI normalized to CD1c^+^ DCs cultured alone with GM-CSF. (C) IL-12p70, IFN-α, and TNF-α in supernatants of overnight cultures were analyzed by ELISA. Black bars, pDCs; white bars, CD1c^+^ DCs; gray bars, CD1c^+^ DCs and pDCs. Results are the mean ± SEM of at least five (A, B) or at least three (C) independent experiments. Significance was determined by repeated measures one-way ANOVA, followed by a post-hoc Dunnett's test (*p < 0.05; **p < 0.01; ***p < 0.001). Only conditions that are significantly different when compared with extra control groups (see Fig. S1) are denoted in the graphs.

We first investigated whether human DC subsets can directly alter each other's activation status. Autologous human CD1c^+^ DCs and pDCs were isolated from peripheral blood and stimulated overnight, either separately (single cultures) or together (co-cultures) at a 1:1 ratio. The total number of DCs was kept constant between the cultures. DCs were stimulated with a specific TLR ligand: CpG oligodeoxynucleotide (ODN) class C (CpG-C) for pDCs, polyinosinic-polycytidylic acid (poly(I:C)) for CD1c^+^ DCs. In the absence of TLR agonists, IL-3 and granulocyte-macrophage colony-stimulating factor (GM-CSF) were added to maintain the survival of pDCs and CD1c^+^ DCs, respectively. Subset-specific maturation status was determined by flow cytometry analysis of the co-stimulatory molecules CD86, CD80, and CD40, the maturation marker CD83, the co-inhibitory molecule programmed death-ligand 1 (PD-L1), C-C chemokine receptor type 7 (CCR7), and major histocompatibility complex (MHC) class I and II molecules. The presence of poly(I:C)-activated CD1c^+^ DCs led to significantly enhanced expression levels of CD86 (*p* = 0.0243), PD-L1 (*p* = 0.0002) and MHC class I (*p* < 0.0001) on unstimulated pDCs (Fig. 1A). Conversely, immature CD1c^+^ DCs significantly upregulated CD86 (*p* = 0.0445), CD80 (*p* < 0.0001), PD-L1 (*p* < 0.0001), and MHC class I (*p* = 0.0014) and slightly downregulated MHC class II (*p* = 0.0414), when co-cultured with CpG-C-activated pDCs (Fig. 1B). When both subsets were stimulated together, using their respective TLR agonists, pDCs showed increased CD86 expression compared to CpG-C-stimulated pDCs cultured separately (*p* = 0.0464), whereas CD1c^+^ DCs showed a trend toward increased PD-L1 expression (*p* = 0.0741). To exclude the possibility that changes in DC maturation status were the result of a combination of stimuli, rather than crosstalk between DC subsets, combinations of stimuli that were used for DC co-cultures were also tested on DC single cultures. Incorporation of these extra control groups did not change the above mentioned results qualitatively (Fig. S1 A and B). Of note, the combination of CpG-C and poly(I:C) did not fully mature CD1c^+^ DCs in both DC single cultures and co-cultures, as co-stimulatory molecules were upregulated less when poly(I:C) was used as the sole stimulus.

Next, cytokine secretion by human blood DCs was examined. Poly(I:C)-stimulated CD1c^+^ DCs secreted IL-12p70, the bioactive form of IL-12, although no detectable levels were reached when a combination of poly(I:C) and CpG-C was used (Fig. 1C, first panel and Fig. S1C). In contrast, CpG-C-stimulated pDCs produced high levels of IFN-α. Interestingly, IFN-α secretion was significantly augmented in DC co-cultures where CpG-C and poly(I:C) were used, compared to CpG-C-stimulated pDCs alone (*p* = 0.0255; Fig. 1C, second panel). Furthermore, both subsets produced tumor necrosis factor (TNF)-α upon TLR ligation. When poly(I:C)-stimulated CD1c^+^ DCs were co-cultured with immature pDCs, there was a trend toward more TNF-α secretion compared to the corresponding DC single cultures (*p* = 0.1537 when compared to poly(I:C)-treated CD1c^+^ DCs alone; Fig. 1C, third panel). These results indicate that TLR triggering of one DC subset induces cross activation of the other subset in culture. In addition, TLR triggering of both DC subsets may induce further changes to their phenotype and functionality, such as increased expression of co-stimulatory molecules and secretion of cytokines.

To assess whether paracrine cytokine stimulation is the main mechanism behind the upregulation of maturation markers observed in the DC co-cultures, immature CD1c^+^ DCs were cultured with different concentrations of recombinant IFN-α or TNF-α, whereas immature pDCs were cultured with different combinations of recombinant TNF-α. IFN-α by itself induced strong upregulation of CD86, CD80, PD-L1, and MHC class I on CD1c^+^ DCs (Fig. S2A), all to a similar extent as can be seen in the DC co-cultures (Fig. 1). Incubation with TNF-α alone induced upregulation of PD-L1 and MHC class I and a slight decrease of CD86 on immature CD1c^+^ DCs (Fig. S2B), whereas at the highest tested concentration, it induced a slight but significant upregulation of CD80 on immature pDCs (*p* = 0.0253; Fig. S2C). Thus, although TNF-α plays a partial role in the crosstalk between pDCs and CD1c^+^ DCs, the cross activation of CD1c^+^ by activated pDCs seems predominantly mediated by IFN-α.

**Figure 2.**
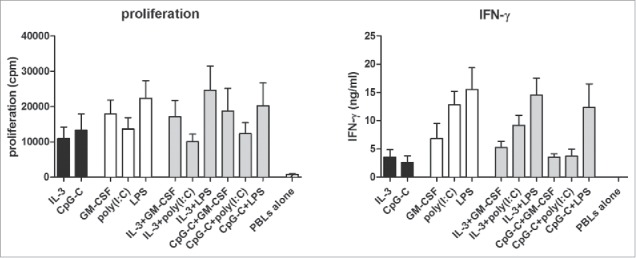
Co-cultures of human CD1c^+^ DCs and pDCs induce effector cell responses in a mixed lymphocyte reaction. After CD1c^+^ DCs and/or pDCs were stimulated overnight with indicated stimuli, they were cultured with allogeneic PBLs at a 10:1 PBL:DC ratio. Proliferation was determined at day 4 by [^3^H]-thymidine incorporation. IFN-γ in supernatants was analyzed at day 3 by ELISA. Black bars, pDCs with PBLs; white bars, CD1c^+^ DCs with PBLs; gray bars, CD1c^+^ DCs and pDCs with PBLs; striped bars, PBLs only. Results are the mean ± SEM of five (first panel) or three (second panel) independent experiments.

### Combined application of CD1c^+^ DCs and pDCs sustains their capacity to activate lymphocytes

Following our observation that CD1c^+^ DCs and pDCs could cross activate each other, we investigated whether this may influence the DCs' ability to activate various immune effector cells. Since poly(I:C) did not optimally mature CD1c^+^ DCs in the presence of CpG-C, stimulation by lipopolysaccharide (LPS) was included as a condition in all further assays. LPS induced strong maturation of mDCs, but not pDCs, while no significant IL-12 production was induced (data not shown). First, a mixed lymphocyte reaction (MLR) assay was performed to determine if DC co-cultures have an effect on the proliferation and cytokine production of PBLs. Expectedly, both CD1c^+^ DCs and pDCs induced proliferation of allogeneic PBLs. However, the combination of DC subsets did not have an additive effect on PBL proliferation (Fig. 2, first panel). Furthermore, both DC subsets induced IFN-γ secretion in the MLR, although higher IFN-γ levels were reached with TLR-stimulated CD1c^+^ DCs than with pDCs (Fig. 2, second panel). Similar to PBL proliferation, DC co-cultures did not yield superior IFN-γ production by PBLs. Collectively, these results clearly demonstrate that human DC subsets, when cultured together, maintain their capacity to induce PBL activation, yet without displaying any synergy.

### Co-cultures of CD1c^+^ DCs and pDCs drive CD4^+^ and CD8^+^ T cell polarization

**Figure 3.**
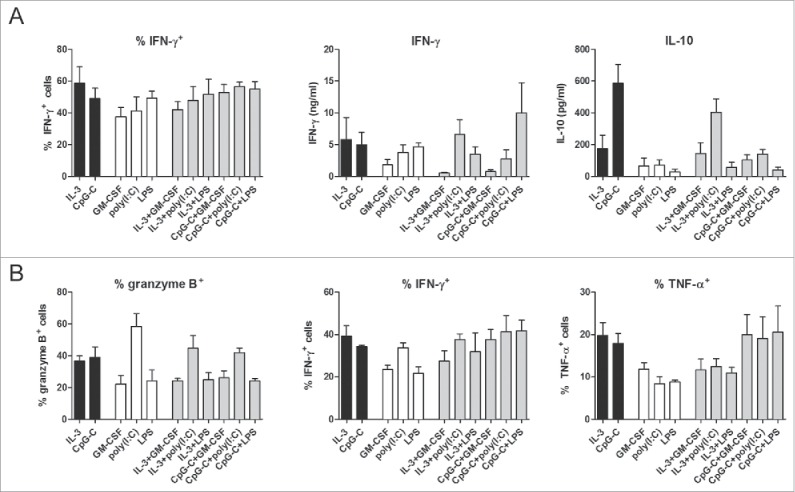
Co-cultures of human CD1c^+^ DCs and pDCs activate and polarize naive CD4^+^ and CD8^+^ T cells. After CD1c^+^ DCs and/or pDCs were stimulated overnight with indicated stimuli, they were cultured with SEB and allogeneic naive CD4^+^ (A) or CD8^+^ (B) T cells at a 4:1 T cell:DC ratio. (A) Resting CD4^+^ T cells were restimulated with PMA/ionomycin in the presence of brefeldin A and intracellular IFN-γ expression was measured by flow cytometry. In parallel, resting CD4^+^ T cells were restimulated with anti-CD3/CD28 beads and after 24 h, IFN-γ and IL-10 in supernatants were analyzed by ELISA. Black bars, pDCs with CD4^+^ T cells; white bars, CD1c^+^ DCs with CD4^+^ T cells; gray bars, CD1c^+^ DCs and pDCs with CD4^+^ T cells. (B) Intracellular granzyme B expression by resting CD8^+^ T cells was measured by flow cytometry. Resting CD8^+^ T cells were restimulated with PMA/ionomycin in the presence of brefeldin A and intracellular IFN-γ and TNF-α expression was measured by flow cytometry. Black bars, pDCs with CD8^+^ T cells; white bars, CD1c^+^ DCs with CD8^+^ T cells; gray bars, CD1c^+^ DCs and pDCs with CD8^+^ T cells. Results are the mean ± SEM of at least five (A, first panel), four (A, second and third panels; B, second and third panels), or three (B, first panel) independent experiments.

DCs are important for the polarization of CD4^+^ T cells into the different T helper cell subsets, as well as the induction of CTLs from naive CD8^+^ T cells. Tumor-specific CD8^+^ T cell responses are critical for the eradication of tumor cells in cancer immunotherapy. In addition, tumor-specific CD4^+^ T cells contribute to CD8^+^ T cell responses and their targeting enhances efficacy of DC vaccination for treatment of melanoma patients.^17,18^ For this purpose, these effector CD4^+^ T cells should exhibit a Th1 phenotype, which is characterized by secretion of IFN-γ.^19^ In sharp contrast, differentiation of CD4^+^ T cells into IL-10-secreting regulatory T cells inhibits adaptive immune responses and thereby promotes tumor immune evasion.^20^ To determine if a combination of human blood DC subsets can efficiently polarize T cells into effector cells that are favorable for cancer immunotherapy, CD1c^+^ DCs and/or pDCs were used to activate allogeneic naive CD4^+^ or CD8^+^ T cells. T cell polarization was determined by analyzing cytokine production profile of the induced T cells. Both DC single cultures and DC co-cultures induced IFN-γ^+^ CD4^+^ T cells (Fig. 3A, first panel). Total IFN-γ secretion in the CD4^+^ T cell cultures differed slightly between the different DC stimuli, but no significant changes were detected for the combination of CD1c^+^ DCs and pDC, compared to the use of either subset alone (Fig. 3A, second panel). In addition, a favorable ratio of IFN-γ to IL-10 was induced in DC co-cultures and IL-10 production in DC co-cultures was lower than in cultures with CpG-C-activated pDCs (Fig. 3A, third panel). These results indicate that the combination of CD1c^+^ DCs and pDCs predominantly drives Th1 polarization, with low induction of regulatory T cells. Finally, CD1c^+^ DCs, pDCs or the combination of both induced granzyme B^+^ CD8^+^ T cells, IFN-γ^+^ CD8^+^ T cells and TNF-α^+^ CD8^+^ T cells, with no significant advantage of DC co-cultures over DC single cultures (Fig. 3B). Thus, combining CD1c^+^ DCs and pDCs does not further augment T cell polarization in comparison to the application of either DC subset alone.

### Co-cultures of CD1c DCs and pDCs promote plasma cell differentiation of activated B cells

**Figure 4.**
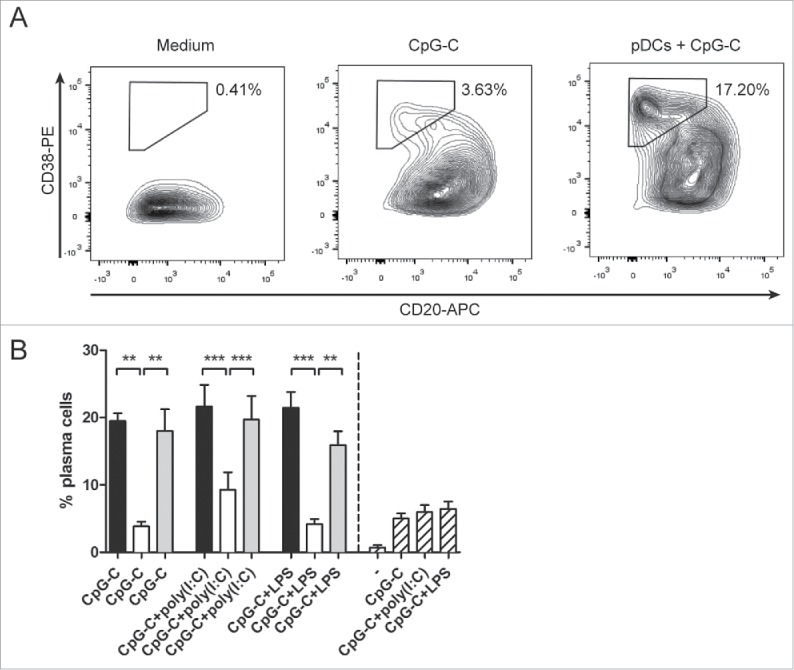
Co-cultures of human CD1c^+^ DCs and pDCs induce plasma cell differentiation. CD1c^+^ DCs and/or pDCs were cultured with autologous B cells in the presence of indicated stimuli. After 7 days, plasma cell differentiation was determined by flow cytometry. (A) Plasma cells were defined as CD38^high^CD20^low^ expressing cells. (B) The percentages of plasma cells in co-cultures with DCs. Black bars, pDCs with B cells; white bars, CD1c^+^ DCs with B cells; gray bars, CD1c^+^ DCs and pDCs with B cells; striped bars, B cells only. Results are the representative or mean ± SEM of three independent experiments. Significance was determined by repeated measures one-way ANOVA, followed by a post-hoc Bonferroni's test (*p < 0.05; **p < 0.01; ***p < 0.001).

Although combined application of pDCs and CD1c^+^ DCs did not have an added value in T cell activation, we investigated whether it has an effect on other immunological components that are essential for tumor eradication. B cells have a crucial role in antitumor immunity as antibody-secreting plasma cells.^21^ Plasma cell differentiation from naive B cells can be promoted by activated pDCs.^22^ We examined if this effect persists in co-cultures of human CD1c^+^ DCs and pDCs. Freshly isolated CD1c^+^ DCs and/or pDCs were incubated together with autologous B cells in the presence of CpG-C and other stimuli for 7 d. CpG-C acts as a stimulus for both pDCs and B cells, and poly(I:C) or LPS was added to stimulate CD1c^+^ DCs. In sharp contrast to CD1c^+^ DCs, activated pDCs enhanced plasma cell differentiation of activated B cells (Fig. 4 A and B). Combining DC subsets did not enhance the plasma cell-inducing capacity of pDCs, which remained significantly higher in comparison to CD1c^+^ DC single cultures (*p* = 0.0095 for cultures stimulated with CpG-C, *p* = 0.0004 for cultures stimulated with CpG-C and poly(I:C), *p* = 0.0043 for cultures stimulated with CpG-C and LPS). Thus, owing to the presence of pDCs, DC co-cultures retain the ability to induce plasma cell differentiation.

### Combining CD1c^+^ DCs and pDCs synergistically enhances NK cell cytotoxicity

**Figure 5.**
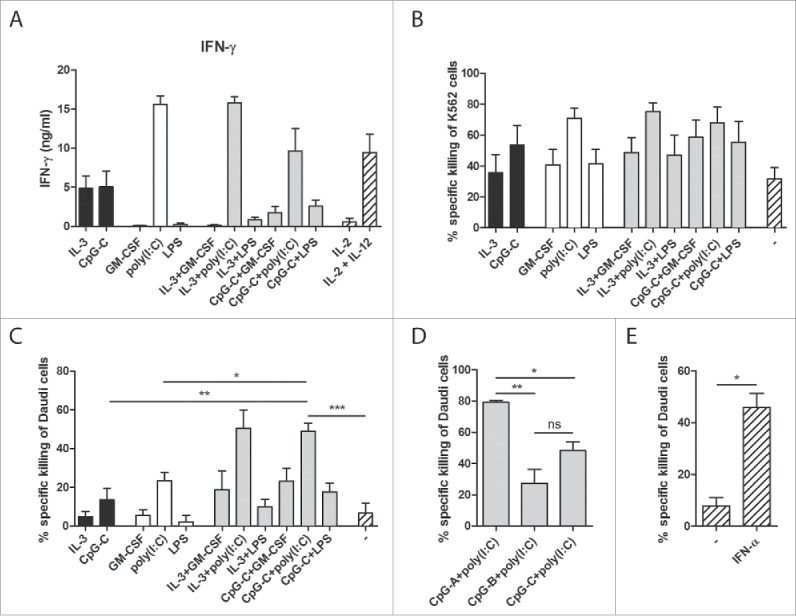
Co-cultures of human CD1c^+^ DCs and pDCs enhance NK cell responses. CD1c^+^ DCs and/or pDCs were cultured overnight with indicated stimuli. (A) Stimulated DCs were subsequently cultured with autologous NK cells at a 1:1 ratio and with IL-2. IFN-γ in 24 h supernatants was analyzed by ELISA. (B–E) Stimulated DCs were cultured with autologous NK cells at a 5:1 NK cell:DC ratio for 24 h. Labeled K562 or Daudi tumor cells were added for the last 4 h of the culture at 5:1 NK cell:target cell ratio. Specific target cell killing was determined by flow cytometry. Black bars, pDCs with NK cells; white bars, CD1c^+^ DCs with NK cells; gray bars, CD1c^+^ DCs and pDCs with NK cells; striped bars, NK cells only. Results are the mean ± SEM of at least four (A, B), five (C), or three (D, E) independent experiments. Significance in (C) was determined by repeated measures one-way ANOVA, followed by a post-hoc Dunnett's test (*p < 0.05; **p < 0.01; ***p < 0.001), and only conditions showing significant differences in all comparisons, are denoted in the graph. Significant differences among experimental conditions in (D) were determined by one-way ANOVA followed by a post-hoc Bonferroni's test (*p < 0.05; **p < 0.01; ***p < 0.001). Significant differences among experimental conditions in (E) were determined by two-tailed Student's *t*-test on paired samples (*p < 0.05; **p < 0.01; ***p < 0.001).

DCs do not only take part in the control of adaptive immunity, but can also interact with innate immune cells such as NK cells. Both IL-12 and IFN-α, cytokines secreted by human blood DCs, are known activators of NK cells.^23^ Therefore, we investigated whether a combination of human CD1c^+^ DCs and pDCs is favorable for the induction of NK cell responses. Both CD1c^+^ DCs and pDCs exerted the ability to enhance IFN-γ secretion by IL-2-activated NK cells, with poly(I:C)-stimulated CD1c^+^ DCs inducing the highest IFN-γ levels (Fig. 5A). Similar trends were seen in NK cell cultures with both DC subsets: conditions in which poly(I:C) was used as DC stimulus induced the highest IFN-γ levels, but not to a higher level than adding CD1c^+^ DCs only.

In addition to IFN-γ secretion, NK cell cytolytic activity, the hallmark of NK functionality, was determined. Following their activation by DCs, NK-killing of either the NK-sensitive K562 tumor cells or the NK-resistant Daudi tumor cells was determined by flow cytometry. Both CpG-C-stimulated pDCs and poly(I:C)-stimulated CD1c^+^ DCs enhanced the killing of K562 and Daudi tumor cells by autologous NK cells (Fig. 5 B and C). When NK cells were activated by the combination of poly(I:C)-stimulated CD1c^+^ DCs and CpG-C-stimulated pDCs, a significant further increase in Daudi cell killing was detected (*p* = 0.0011 when compared to CpG-C-activated pDCs and *p* = 0.0115 when compared to poly(I:C)-activated CD1c^+^ DCs; Fig. 5C). To exclude the possibility that this was merely an effect of the combination of CpG-C and poly(I:C), both stimuli were also tested on DC single cultures. When compared to DC single cultures stimulated with both CpG-C and poly(I:C), the increase in Daudi cell killing in the DC co-cultures remained significant (*p* = 0.0010 when compared to pDCs and *p* = 0.0002 when compared to CD1c^+^ DCs; Fig. S3).

In an attempt to investigate the mechanism behind the observed synergistic effect on NK-cytotoxicity, other CpG classes were used to stimulate the DCs. Although CpG-A mainly induces the secretion of high IFN-α levels by pDCs, CpG-B mainly elevates the expression of co-stimulatory molecules.^24^ As expected, DC co-cultures stimulated with CpG-A secreted more IFN-α than those stimulated with CpG-C, whereas stimulation with CpG-B did not lead to detectable levels of IFN-α (Fig. S4). When CpG-A was used as stimulus in the DC co-cultures instead of CpG-C, higher levels of NK-mediated Daudi cell killing were achieved (*p* = 0.0248; Fig. 5D). In contrast, the use of CpG-B resulted in less killing, although the difference was not statistically significant (*p* = 0.1603). Furthermore, addition of recombinant IFN-α to NK cells alone also increased their cytotoxicity (*p* = 0.0237; Fig. 5E). These results hint toward a role of IFN-α in the synergistic enhancement of NK cell cytotoxicity in co-cultures of human CD1c^+^ DCs and pDCs, which is consistent with the observed increase in IFN-α production in the DC co-cultures.

In summary, human blood CD1c^+^ DCs and pDCs could cross activate each other, while combined TLR-stimulation of both subsets induced further changes, including increased CD86 expression on pDCs and enhanced IFN-α production. These crosstalk-mediated effects did not affect the DCs' ability to induce T cell polarization, but did augment NK cell-mediated killing of an NK-resistant tumor cell line. Furthermore, co-application of CD1c^+^ DCs and pDCs allowed for complementation of subset-specific effector functions, such as induction of plasma cell differentiation from B cells and secretion of high levels of IFN-γ by PBLs and NK cells.

### Discussion

Naturally occurring DCs might become a promising alternative to monocyte-derived DCs for use in DC-based cancer immunotherapy.^3^ Our group previously conducted the first clinical trials with *ex vivo* stimulated and TAA-pulsed primary human DCs in metastatic melanoma patients and demonstrated the safety, feasibility and proof of principle of this approach.^15,16^ Overall survival of patients that received a pDC vaccine was greatly increased compared to matched controls and in 7 out of 15 patients an increase in TAA-specific CD8^+^ T cells was detected after vaccination.^15^ Of 14 patients that received a CD1c^+^ DC vaccine, 4 showed long-term progression-free survival, which correlated with multifunctional CD8^+^ T-cell responses in 3 of these patients.^16^ Others have shown that primary DCs might also be used for treatment of cancers other than melanoma. Vaccination with TAA-pulsed CD1c^+^ DCs for advanced stage metastatic prostate cancer was considered feasible and safe.^14^ These positive results prompted us to investigate whether a combination of primary DC subsets can further enhance immunological responses.

A drawback of the use of primary DCs for immunotherapy is that the number of DCs that can be isolated from peripheral blood is relatively low. Combining CD1c^+^ DCs and pDCs for vaccination purposes would mean that a larger number of DCs can be adoptively transferred, thereby potentially enhancing immune responses. In addition, CD1c^+^ DCs and pDCs are functionally distinct and may interact differently with adaptive and innate immune cells, suggesting that they might be able to complement each other's effector functions. Furthermore, several *in vitro* and *in vivo* studies have demonstrated that direct crosstalk between DC subsets can take place both in the human and murine setting and is mediated by soluble and contact-dependent mechanisms.^25,26^ Evidence for synergy between mouse DC subsets was found in a study by Lou *et al.*, which showed that immunization of mice with a mixture of mDCs and activated pDCs improved antitumor responses, compared to immunization with either subset alone.^27^

In this study, we compared co-cultures of CD1c^+^ DCs and pDCs to single cultures of either DC subset for their phenotype, cytokine secretion and ability to induce effector cell responses. The total amount of DCs was kept the same in DC single cultures and DC co-cultures, to exclude cell number-dependent effects. TLR agonists were added to mature one particular DC subset: TLR-3 agonist poly(I:C) for CD1c^+^ DCs and TLR-9 agonist CpG-C for pDCs. In line with previous findings,^28,29^ we found that mature CD1c^+^ DCs can activate immature pDCs and *vice versa*. This cross activation effect could therefore also play a role during DC vaccination therapy, where infused mature DCs might propagate initial induced immune responses by maturing host DCs.

One of the issues that needs to be considered when using CD1c^+^ DCs and pDCs into a combined vaccine is whether these subsets should be maturated separately or together. Based on the findings in this manuscript, should only one TLR agonist specific to one of the subsets be used, then it is preferable to stimulate the CD1c^+^ DCs and pDCs together, so that the mature subset can mediate cross activation of the other subset. Also in the *in vivo* study by Lou et al., maturation of pDCs in the presence of mDCs was crucial, as a mixture of mDCs and separately activated pDCs did not result in enhanced antigen-specific CD8^+^ T cell responses.^27^ Since the mDCs in this model were not stimulated by a TLR agonist, the observed synergistic effect can be explained by the cross activation of the immature mDCs by mature pDCs prior to vaccination. In an ideal DC vaccine, however, both subsets should be fully activated, in order to induce maximal immunological responses. Here, we have shown that combined maturation of human blood DC subsets using TLR agonists can also affect their phenotype and functionality. pDCs cultured with CD1c^+^ DCs in the presence of poly(I:C) and CpG-C, upregulated CD86, compared to pDCs receiving similar stimuli in the absence of CD1c^+^ DCs. However, although crosstalk between DC subsets leads to the upregulation of co-stimulatory molecules, it also leads to upregulation of PD-L1. While engagement of co-stimulatory molecules enhances T cell activation, binding of PD-L1 to the PD-1 receptor on T cells provides an inhibitory signal that suppresses T cell activation.^30^ Besides changing their expression of surface molecules, co-cultures of CD1c^+^ DCs and pDCs stimulated with poly(I:C) and CpG-C produced more IFN-α than single cultures of stimulated pDCs. The difference was significant even though the absolute number of pDCs, the main producers of IFN-α, in the DC co-cultures was half of the amount present in the DC single cultures. IFN-α can, among other effects, induce Th1 skewing of naive CD4^+^ T cells,^12^ which in turn contributes to the antitumor immune response.

To determine the overall effect of CD1c^+^ DC-pDC crosstalk on T cell responses, we cultured stimulated DCs with naive CD4^+^ T cells or naive CD8^+^ T cells. No significant changes in cytokine production by the T cells were observed for the combination of DC subsets compared to the use of single DC subsets. Hence, combining CD1c^+^ DCs with pDCs does not seem to negatively alter their ability to induce CD4^+^ and CD8^+^ T cell responses. However, the observed upregulation of PD-L1 in the DC co-cultures, suggests that certain DC vaccines might benefit from the combination with blocking antibodies directed against PD-L1 and/or PD-1. Thus, a combination of DC subsets may synergistically enhance T cell responses, when used in conjunction with these checkpoint inhibitors.

Besides efficiently priming naive T cells, DCs possess the ability to activate other immune effector cells, including B cells and NK cells. NK cells are critical for the antitumor immune response, but the role of B cells in tumor immunity is controversial.^31-37^ Nevertheless, it has recently been reported that antibodies directed against tumor cells can promote antitumor responses by facilitating enhanced uptake of tumor antigens by DCs. ^38^ In addition, tumor cell-specific antibodies can mediate complement dependent and cell dependent cytotoxicity.^21^ Antibodies are mainly produced by plasma cells, which develop from activated B cells. We have shown that pDCs are superior to CD1c^+^ DCs at enhancing plasma cell differentiation from activated B cells. In contrast, CD1c^+^ DCs outperformed pDCs at enhancing IFN-γ production in both an MLR assay and in culture with activated autologous NK cells. The advantage of one particular DC subset was retained when the subsets were combined. Thus, human blood mDCs and pDCs can complement each other's effector functions, even when cultured together.

CTLs can detect and lyse transformed cells in an antigen-dependent manner, by recognizing neo-antigens or overexpressed self-antigens. DC vaccine-based immunotherapy has therefore historically focused at inducing strong CD8^+^ T cell responses. However, the importance of DC interactions with innate cells for clinical outcome is now becoming more appreciated.^39^ NK cells form the body's main line of defense against tumor cells that downregulate MHC class I to evade detection by CTLs. Besides reacting to a loss of inhibitory ligands, NK cells possess activating receptors that recognize stress-induced ligands on tumor cells. NK cells do not necessarily require prior activation to lyse target cells and in our experiments they could efficiently kill K562 tumor cells. Still, addition of poly(I:C)-activated CD1c^+^ DCs, CpG-C-activated pDCs or both to the culture enhanced NK cell cytotoxicity. However, other tumor cells may be more resistant to NK cell-mediated killing. In order to lyse such target cells, NK cell do require prior stimulation. In line with previous findings,^40^ we found that NK cells alone only induce minor killing of Daudi tumor cells, but culture of NK cells with poly(I:C)-activated CD1c^+^ DCs or CpG-C-activated pDCs enhanced their killing potential. Moreover, the combination of both DC subsets further increased the killing. This synergistic effect is most likely mediated by the increased amount of IFN-α produced by the DC co-cultures, as replacement of CpG-C by CpG-A to mature DCs further enhanced NK-mediated Daudi cell killing, while use of CpG-B instead lowered the killing. No synergistic increase in NK-mediated killing could be detected in our assays with K562 tumor cells, most likely because optimal killing is already achieved when one of the DC subsets is added.

Our results indicate that combining CD1c^+^ DCs and pDCs for therapeutic purposes can have beneficial effects. Furthermore, stimulation of CD1c^+^ DCs and pDCs together allows for direct crosstalk between the subsets, which can alter their phenotype and functionality. However, using multiple stimuli to facilitate maturation of DC subsets in co-culture may have additional, non-crosstalk-related effects. CD1c^+^ DCs did not optimally mature in the presence of both poly(I:C) and CpG-C: the increase in co-stimulatory molecules was lower than when CpG-C was absent and no detectable levels of IL-12p70 were produced. This can have consequences for their effector function, as DC co-cultures that received poly(I:C) and CpG-C induced lower IFN-γ levels in both an MLR assay and in culture with autologous NK cells, than when poly(I:C) and IL-3 were used as stimuli. The inhibitory effect of CpG ODN on poly(I:C)-induced DC maturation has been described before for monocyte-derived DCs.^41^ We therefore took along LPS as a stimulus for CD1c^+^ when they were subsequently cultured with effector cells. Although CD1c^+^ DCs stimulated with this TLR-4 agonist express high levels of co-stimulatory molecules, they do not secrete detectible levels of IL-12p70.^8^ This is likely the reason why LPS-stimulated CD1c^+^ DCs were unable to enhance IFN-γ production or tumor cell killing by autologous NK cells. Ideally, the DC subsets should be stimulated in such a way that pDCs secrete high levels of IFN-α and CD1c^+^ DCs secrete high levels of bioactive IL-12. While CpG ODN inhibits poly(I:C)-induced DC maturation, a combination of other TLR agonists may actually enhance DC activation.^42^ Alternatively, both DC subsets can be stimulated with a single TLR agonist. Our group recently established a clinical-grade stimulus that can activate both CD1c^+^ DCs and pDCs in the form of stabilized RNA complexes.^43^ The use of such a stimulus would be optimal to exploit the advantages of both DC subsets in a clinical setting.

Taken together, a combination of CD1c^+^ DCs and pDCs allows for complementation or even enhancement of each other's effector functions. Hence, combining different human blood DC subsets holds promise for improvement of current DC-based anticancer vaccines.

## Materials and methods

### Cell isolation

Primary cells were isolated from buffy coats of healthy individuals (Sanquin) after informed consent and according to institutional guidelines. Peripheral blood mononuclear cells (PBMCs) were isolated by Ficoll density centrifugation (Lymphoprep; Axis-Shield PoC). B cells were isolated from PBMCs with a pan B cell isolation kit (Miltenyi Biotec, 130-101-638). CD1c^+^ DCs were isolated from PBMCs with a CD1c^+^ DC isolation kit (Miltenyi Biotec, 130-090-506), after depleting CD14^+^ cells with anti-CD14-conjugated microbeads (Miltenyi Biotec, 130-050-201). PBLs were prepared by depleting PBMCs of monocytes with anti-CD14-conjugated microbeads or via adherence to plastic culture flasks. pDCs were isolated from PBLs with a pDC isolation kit (Miltenyi Biotec, 130-090-532). Naive CD4^+^ T cells were isolated by enriching PBLs for CD4^+^ T cells with a CD4^+^ T cell isolation kit (Miltenyi Biotec, 130-096-533) and subsequently depleting CD45RO^+^ memory T cells with anti-CD45RO-PE (Dako, R084301) and anti-PE-conjugated microbeads (Miltenyi Biotec, 130-048-801). Naive CD8^+^ T cells and NK cells were isolated from PBLs with a naive CD8^+^ T cells isolation kit (Miltenyi Biotec, 130-093-244) and an NK cell isolation kit (Miltenyi Biotec, 130-092-657), respectively. Cell purity was up to 98% as assessed by double staining for CD1c/CD11c, BDCA2/CD123, CD4/CD45RA, CD8/CD45RA, CD56/CD3, and CD19/CD3 for CD1c^+^ DC, pDC, naive CD4^+^ T cell, naive CD8^+^ T cell, NK cell, and B cell fractions, respectively.

All isolated human peripheral cells were cultured in X-VIVO 15 medium (Lonza, BE04-418Q) supplemented with 2% human serum (Sanquin).

### DC activation

The following TLR ligands were used for DC stimulation: 5 μg/mL CpG-C (ODN M362; Enzo Life Sciences, ALX-746-004), 5 μg/mL CpG-A (ODN 2216; Enzo Life Sciences, ALX-746-005) or 5 μg/mL CpG-B (ODN 2006; Enzo Life Sciences, ALX-746-006) for pDCs and 20 μg/mL poly(I:C) (Enzo Life Sciences, ALX-746-021) or 1 μg/mL LPS (Sigma-Aldrich, L4391-10×1MG) for CD1c^+^ DCs. In the absence of their respective TLR ligands, 10 ng/mL IL-3 (CellGenix, 1002-050) was added to increase pDC viability and 450 U/mL GM-CSF (CellGenix, 1012–050) was added for CD1c^+^ DCs. Where indicated, DCs were also incubated in the presence of increasing concentrations of IFN-α-2a (Roferon®-A; Roche) or TNF-α (CellGenix, 1006).

### Cell lines

The human NK-sensitive K562 tumor cell line was obtained from the American Type Culture Collection (ATCC, catalog number: K-562 ATCC CCL-243). The human NK-resistant Daudi tumor cell line was obtained from ATCC (catalog number: Daudi ATCC CCL-213) and kindly provided to us by the laboratory of Prof Kris Thielemans (Free University of Brussels, Brussels, Belgium). Both cell lines were cultured in Iscove's modified Dulbecco's medium (Invitrogen, 12440053) supplemented with 10% fetal bovine serum (Greiner Bio-One) and 1% antibiotic antimycotic (Gibco, 15240062).

### Phenotype and cytokine secretion of stimulated DCs

CD1c^+^ DCs and pDCs were incubated overnight at 37°C with different stimuli in a 96-well round-bottom plate, either separate or together at a 1:1 ratio, with each well containing equal cell numbers (50 × 10^3^ cells in 100 μL). After overnight culture, supernatants were taken and cells were stained with the following primary monoclonal antibodies: anti-human leukocyte antigen (HLA)-ABC-APC (BD Biosciences, 555555), anti-HLA-DR/DP/DQ-FITC (BD Biosciences, 555558), anti-CD80-PE (BD Biosciences, 557227), anti-CD83-FITC (BD Biosciences, 556910), anti-CD83-APC (BD Biosciences, 551073), anti-CD86-PE (BD Biosciences, 555658), anti-PDL-1-PE (BD Biosciences, 557924), anti-CD40-PE (Beckman Coulter, PN IM1936U), anti-CCR7-PE (Miltenyi Biotec, 130-093-621). Anti-CD11c-PE (BD Biosciences, 333149) or anti-CD123-APC (Miltenyi Biotec, 130-090-901) primary monoclonal antibodies were used to distinguish between DC subsets in co-cultures. Samples were measured on a FACSCalibur or FACSVerse (BD Biosciences) and analyzed by FlowJo software (TreeStar, Inc.). The results are depicted as geometric mean fluorescence intensity (MFI) normalized to the negative control. Supernatants were analyzed for IL-12p70 (Thermo Fisher Scientific, M122), TNF-α (eBioscience, 88-7346-88) and IFN-α (Bender Medsystems, BMS216MST) by sandwich ELISA.

### Mixed lymphocyte reaction

CD1c^+^ DCs and pDCs were incubated overnight at 37°C with different stimuli in a 96-well round-bottom plate, either separate or together at a 1:1 ratio, with each well containing equal cell numbers. After overnight culture, DCs were washed with culture medium to remove residual stimuli before subsequent culture with effector cells.

The ability of DCs to induce T cell proliferation and cytokine production was tested in an MLR, using 100 × 10^3^ allogeneic PBLs that were added to the DCs at a 10:1 ratio (PBL:DC) in 200 μL and incubated for 4 d at 37°C. On day 3, supernatants were taken and analyzed for IFN-γ (Thermo Fisher Scientific, M700A) by sandwich ELISA. Cellular proliferation was analyzed by measuring overnight [^3^H]-thymidine (1 µCi/well; MP Biomedicals, 2407005) incorporation on day 4 with a β counter.

### CD4^+^ T cell polarization

CD1c^+^ DCs and pDCs were stimulated as above and co-cultured with allogeneic naive CD4^+^ T cells at a 4:1 ratio (T cell:DC) in the presence of 10 pg/mL Staphylococcal enterotoxin B (SEB; Sigma-Aldrich, S4881). Proliferating cells at day 5 were transferred to 48-well plates and refreshed every other day with medium supplemented with 20 U/mL IL-2 (Proleukin®, Chiron) until cells were resting around day 11. 100 × 10^3^ resting T cells were restimulated by anti-CD3/CD28 beads (1 bead per T cell; Gibco, 11131D) in 100 μL and after 24 h the levels of IL-10 (eBioscience, 88-7106-77) and IFN-γ were determined in supernatants by sandwich ELISA. In parallel, resting CD4^+^ T cells were restimulated by phorbol 12-myristate 13-acetate (PMA; Merck Millipore, 524400)/ionomycin (Sigma-Aldrich, I0634) in the presence of brefeldin A (Sigma-Aldrich, B6542) for 6 h, after which cells were fixed with 4% paraformaldehyde (Merck Millipore, 104005), permeabilized with saponin (Sigma-Aldrich, 84510) and stained with anti-IFN-γ-PerCp-Cy5.5 (BD Biosciences, 560704). Samples were measured on a CyAn ADP (Beckman Coulter).

### Naive CD8^+^ T cell activation

CD1c^+^ DCs and pDCs were stimulated as above and co-cultured with allogeneic naive CD8^+^ T cells at a 4:1 ratio (T cell:DC) in the presence of 1 pg/mL SEB. Expression of granzyme B by CD8^+^ T cells was measured on day 5 by intracellular staining using anti-GrB-PE antibody (Sanquin, M2289). To detect intracellular cytokine production, CD8^+^ T cells were restimulated on day 6 with PMA/ionomycin in the presence of brefeldin A for 6 h and after fixation and permeabilization, stained with anti-IFN-γ-PerCp-Cy5 and anti-TNF-α-PE (Biolegend, 502909). Samples were measured on a CyAn ADP.

### Plasma cell differentiation

In contrast to the other effector cell assays, B cells were co-cultured directly with freshly isolated DCs. Autologous B cells were incubated with or without DCs at a 10:1 ratio (B cell:DC) in the presence of different stimuli, for 7 d at 37°C. DCs were CD1c^+^ DCs, pDCs or a combination of both at a 1:1 ratio, with each well containing equal total DC numbers. After 7 d, B cells were stained with CD20-APC (eBioscience, 17-0209-42) and CD38-PE (ImmunoTools, 21270384) or CD38-PerCP-Cy5.5 (BD Biosciences, 551400) and a viability dye (Fixable Viability Dye eFluor 780 or 506; eBioscience, 65-0865-18 or 65-0866-18) and measured on a FACSVerse. Plasma cells were defined as CD38^high^CD20^low^ expressing cells.

### NK cell activation and cytotoxicity

CD1c^+^ DCs and pDCs were stimulated as mentioned for the MLR assay, co-cultured with 100 ×10^3^ autologous NK cells at a 1:1 ratio in 200 μL and incubated for 24 h at 37°C in the presence of 100 U/mL IL-2. NK cells cultured without DCs, but with IL-2 or IL-2 and 1 ng/mL IL-12p70 (BD Biosciences, 554613), were used as controls. Supernatants were taken after 24 h and analyzed for IFN-γ by sandwich ELISA.

For cytotoxicity assays, CD1c^+^ DCs and pDCs were stimulated as above, co-cultured with autologous NK cells at a 5:1 ratio (NK:DC) and incubated for 24 h at 37°C. NK cells cultured without DCs and with or without 10 ng/mL IFN-α-2a (Roferon®-A; Roche) were used as reference. Target cells (K562 or Daudi) were labeled with PKH67 green fluorescent cell linker (Sigma-Aldrich, MIDI67-1KT) according to manufacturer's instruction and added to the NK cell cultures for the last 4 h of the culture at an NK cell:target cell ratio of 5:1. Target cells cultured without NK cells were used as negative control. Cell death was determined after staining with propidium iodide (PI; Biolegend, 1056–1) and annexin-V-APC (BD Biosciences) in annexin-V binding buffer (BD Biosciences, 550475). Samples were measured on a FACSVerse or FACSAria II (BD Biosciences). Specific killing of target cells was calculated using the formula: %killing = 100 − [(%Annexin−V^−^PI^−^ target cells with NK cells/%Annexin−V^−^PI^−^ target cells without NK cells) × 100].^44^

To assess IFN-α secretion by DC co-cultures during the cytotoxicity assay, CD1c^+^ DCs and pDCs were stimulated as above (20 ×10^3^ cells in 100 μL). After 12 h of overnight culture, supernatants were taken, and DCs were washed with culture medium to remove residual stimuli before subsequent culture of 24 h, after which supernatants were again taken. As a control, supernatants were also taken of DCs cultured for 36 h without washing.

### Statistical analysis

GraphPad Prism software (v6.01) was used for statistical analysis. For multiple comparisons, repeated measures one-way ANOVA was performed, followed by post-hoc Dunnett's test when comparisons were made with one group, or Bonferroni's test when comparisons were made with all groups, to detect statistical significant differences between groups. For single comparisons, paired Student's *t*-tests were performed to detect statistical significant differences between groups. *p*-Values < 0.05 were considered statistically significant (*p < 0.05; **p < 0.01; ***p < 0.001).

## Supplementary Material

KONI_A_1227902_s02.docx
