# Supplementary material for: Human blood myeloid and plasmacytoid dendritic cells cross activate each other and synergize in inducing NK cell cytotoxicity
Source: Oncoimmunology. 2016 Sep 2;5(10):e1227902. doi: 10.1080/2162402X.2016.1227902 (PMC5087293; doi:10.1080/2162402X.2016.1227902)
Supplement: KONI_A_1227902_s02.docx [file koni-05-10-1227902-s001.docx]

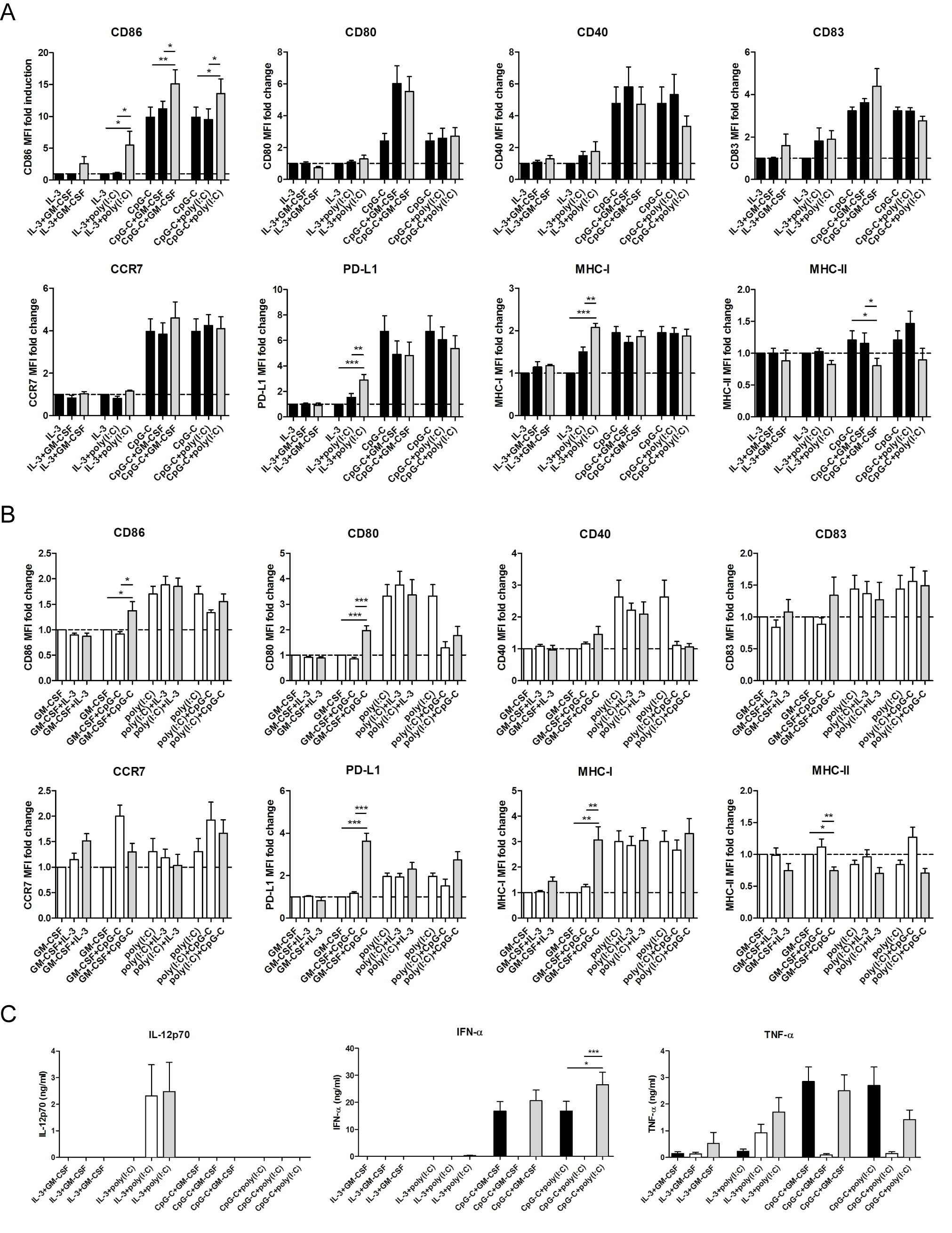


**Supplementary Figure 1.** **Human CD1c^+^ DCs and pDCs cross activate each other.** CD1c^+^ DCs and pDCs were cultured overnight, either separately or together, in the presence of indicated stimuli. Maturation was checked on the specific DC subsets within the coculture. **A**) Relative expression of CD86, CD80, CD40, CD83, PD-L1, CCR7 and MHC class I and II on pDCs in geometric MFI normalized to pDCs cultured alone with IL-3. **B**) Relative expression of CD86, CD80, CD40, CD83, PD-L1, CCR7 and MHC class I and II on CD1c^+^ DCs in geometric MFI normalized to CD1c^+^ DCs cultured alone with GM-CSF. **C**) IL-12p70, IFN-α and TNF-α in supernatants of overnight cultures were analyzed by ELISA. Black bars, pDCs; white bars, CD1c^+^ DCs; gray bars, CD1c^+^ DCs and pDCs. Results are the mean ± SEM of at least 5 (A, B) or at least 3 (C) independent experiments. Significance was determined by repeated measures one-way ANOVA, followed by a post hoc Dunnett’s test (^*^*P* < 0.05; ^**^*P* < 0.01; ^***^*P* < 0.001). Only conditions showing significant differences in all comparisons, are denoted in the graphs.

**
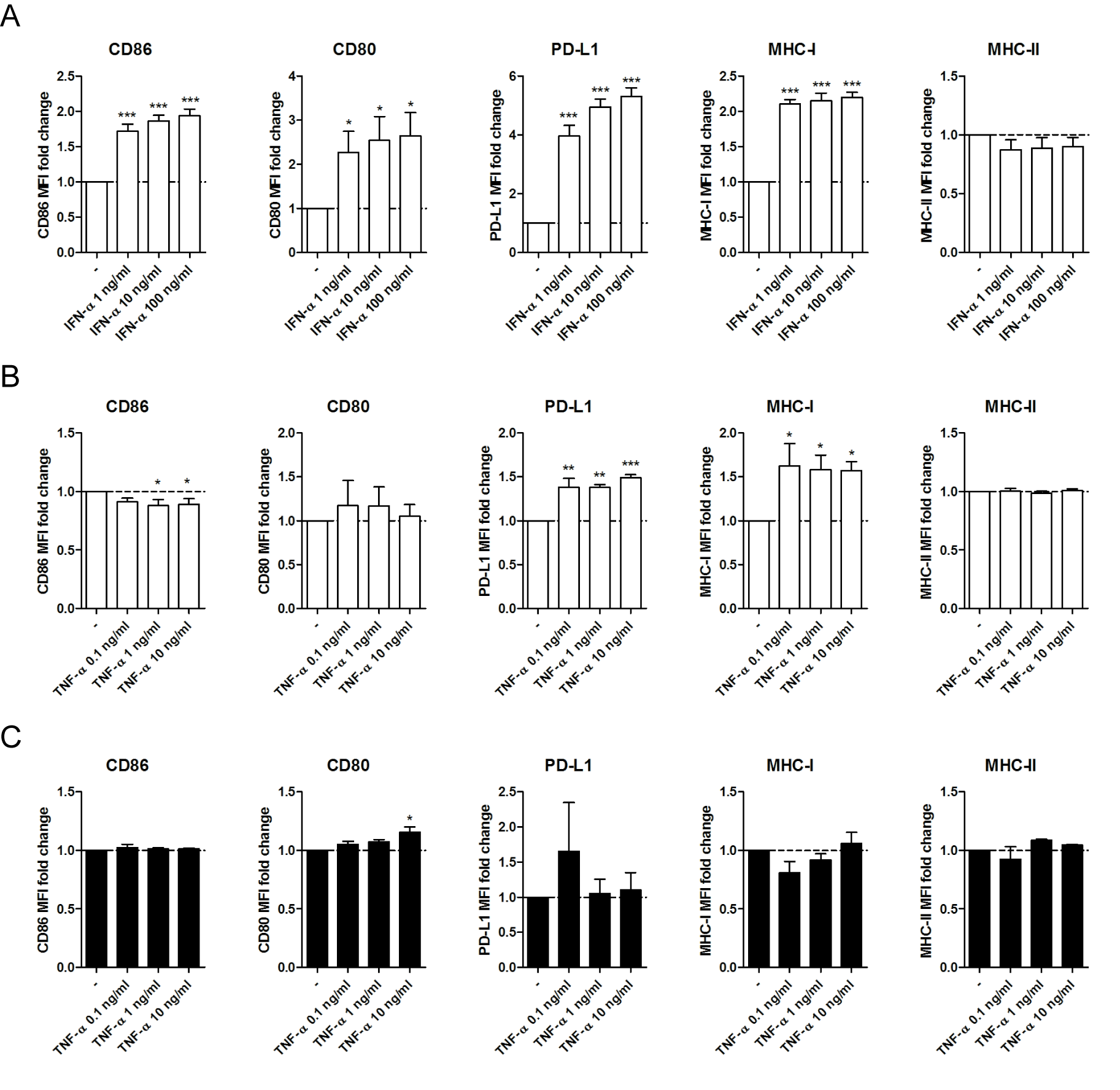
**

**Supplementary Figure 2.** **IFN-α and TNF-α mediate cross activation between CD1c^+^ DCs and pDCs.** CD1c^+^ DCs were cultured overnight in the presence of GM-CSF and increasing concentrations of recombinant IFN-α (A) or TNF-α (B), while pDCs were cultured overnight in the presence of IL-3 and increasing concentrations of recombinant TNF-α. **A, B)** Relative expression of CD86, CD80, PD-L1 and MHC class I and II on CD1c^+^ DCs in geometric MFI normalized to CD1c^+^ DCs cultured with GM-CSF only. **C**) Relative expression of CD86, CD80, PD-L1 and MHC class I and II on pDCs in geometric MFI normalized to pDCs cultured with IL-3 only. White bars, CD1c^+^ DCs; black bars, pDCs. Results are the mean ± SEM of 3 independent experiments. Significance was determined by repeated measures one-way ANOVA, followed by a post hoc Dunnett’s test (^*^*P* < 0.05; ^**^*P* < 0.01; ^***^*P* < 0.001).


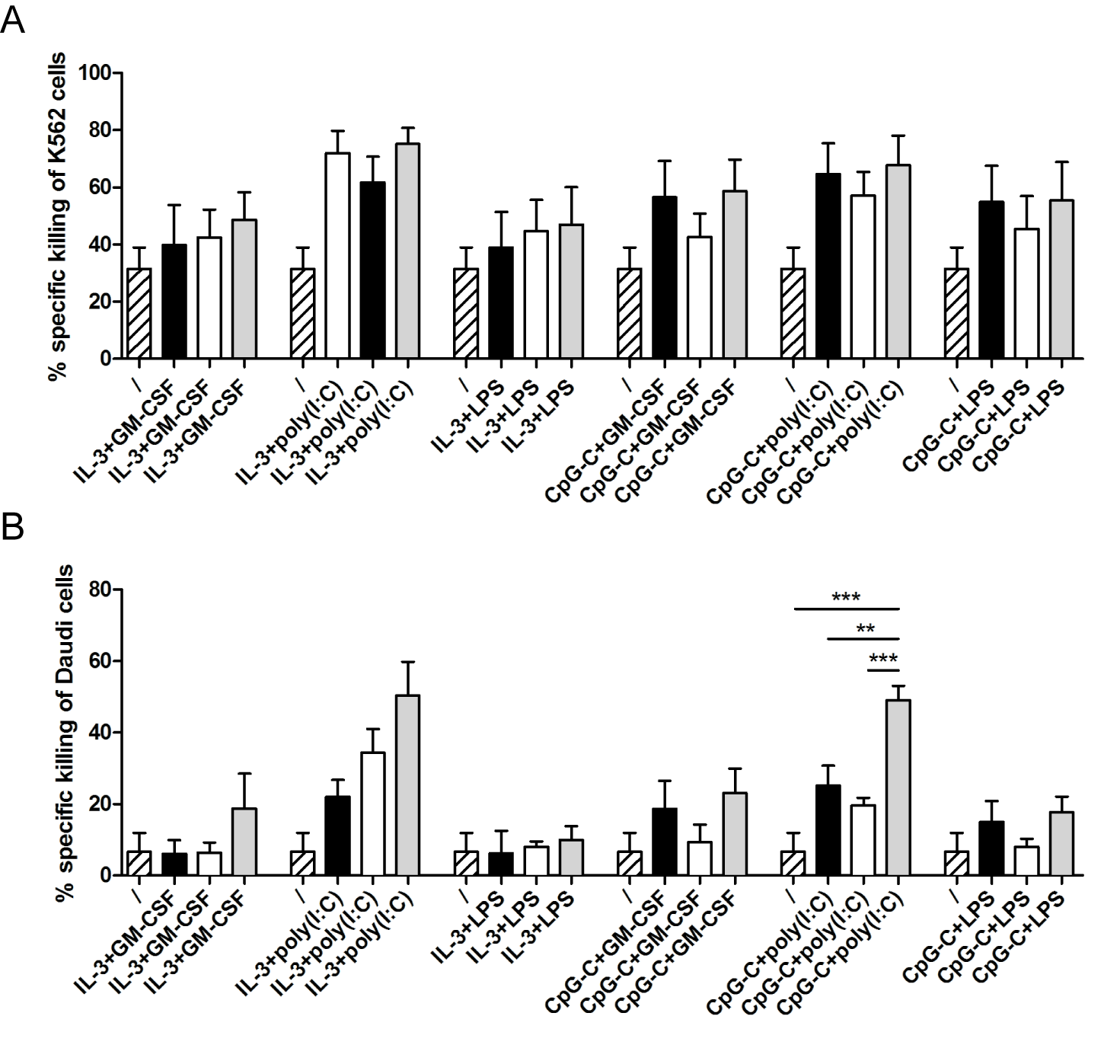


**Supplementary Figure 3.** **Cocultures of human CD1c^+^ DCs and pDCs enhance NK cell responses.** CD1c^+^ DCs and/or pDCs were cultured overnight with indicated stimuli. DCs were subsequently cultured with autologous NK cells at a 5:1 NK cell:DC ratio for 24 hours. Labeled K562 (A) or Daudi (B) tumor cells were added for the last 4 hours of the culture at 5:1 NK cell:Daudi cell ratio. Specific target cell killing was determined by flow cytometry. Striped bars, NK cells only; black bars, pDCs with NK cells; white bars, CD1c^+^ DCs with NK cells; gray bars, CD1c^+^ DCs and pDCs with NK cells. The NK cells only condition is used in multiple comparisons. Results are the mean ± SEM of 4 (A) or 5 (B) independent experiments. Significance was determined by repeated measures one-way ANOVA, followed by a post hoc Dunnett’s test (^*^*P* < 0.05; ^**^*P* < 0.01; ^***^*P* < 0.001). Only conditions showing significant differences in all comparisons, are denoted in the graphs.

**
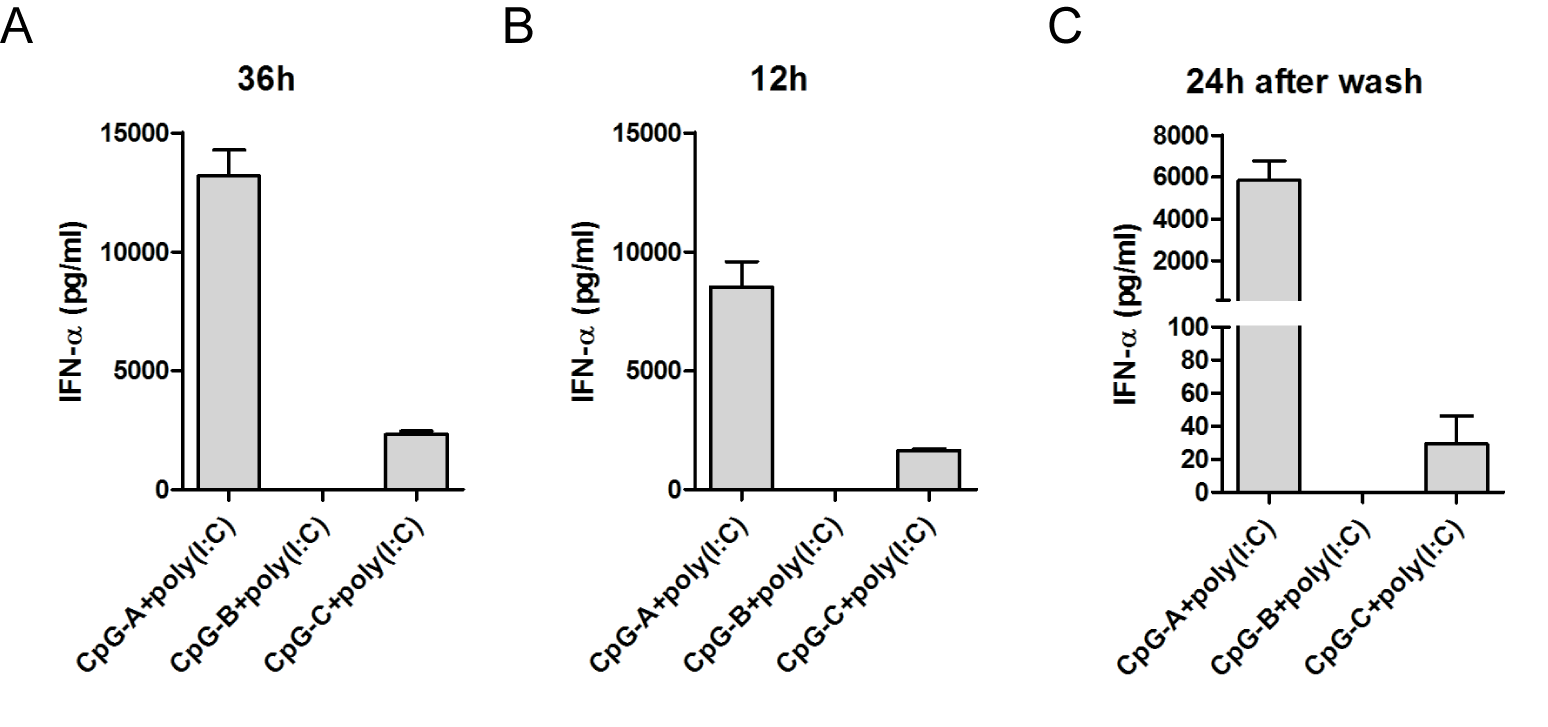
**

**Supplementary Figure 4.** **Cocultures of human CD1c^+^ DCs and pDCs secrete different levels of IFN-α when stimulated with different types of CpG.** CD1c^+^ DCs and pDCs were cultured together at similar concentrations as in the NK cell cytotoxicity assays, with indicated stimuli. Supernatants were taken after 36 hours (A), or 12 hours (B) of culture. Alternatively, when DC cultures were washed following 12 hours of overnight culture, supernatants were taken after 24 hours of subsequent culture (C). IFN-α in supernatants was analyzed by ELISA.
